# Methacrylated gelatin-based conductive self-healing hydrogels: a dual-scale approach for micro- and macro-sized soft materials

**DOI:** 10.1039/d5lp00406c

**Published:** 2026-03-20

**Authors:** Didem Aycan, Maria Regato-Herbella, Fereydoon Taheri, Angeles De la Cruz-García, Sebastian Weber, Neslihan Alemdar, Christine Selhuber-Unkel

**Affiliations:** a Institute for Molecular Systems Engineering and Advanced Materials (IMSEAM), Heidelberg University Heidelberg Germany didem.aycan@marmara.edu.tr; b Department of Chemical Engineering, Marmara University İstanbul Turkey

## Abstract

Soft robotic microsystems, inspired by the flexibility of biological structures, have gained significant research interest due to their ability to navigate complex environments with high adaptability. Electroconductive hydrogels (ECHs) have emerged as promising materials for these systems, offering intrinsic softness, biocompatibility, and electrical conductivity. Here, we present an electroconductive hydrogel with multifunctionality developed using a dual-component conductive strategy, incorporating polyaniline (PANI)–silver (Ag) nanoparticles into a methacrylated gelatin (GelMa) network. The hydrogel was fabricated at two different length scales using complementary fabrication techniques. UV crosslinking was employed to produce macroscale hydrogels, while two-photon lithography was used to demonstrate the feasibility of fabricating microscale structures from the same material system. In addition to their structural versatility, the hydrogels exhibited self-healing behavior that enables autonomous recovery of both mechanical and electrical functionalities after damage, which is important for long-term operation in dynamic environments. Comprehensive characterization, including morphological, electrical, mechanical, and biological tests, confirmed the conductivity, cytocompatibility, and tunable mechanical properties of the hydrogel. The results suggest that this biopolymer-based, electroconductive hydrogel with self-healing ability is a highly promising candidate for next-generation soft robotic systems, offering a durable, adaptable, and bio-integrated solution for further soft robotic applications at both macro- and micro-scales.

## Abstract 2

Multifunctional GelMa/PANI–AgNP hydrogels exhibiting electrical conductivity, self-healing and cytocompatibility were developed. While macro-scale properties were systematically characterized, micro-scale fabrication feasibility was demonstrated.

## Graphical abstract

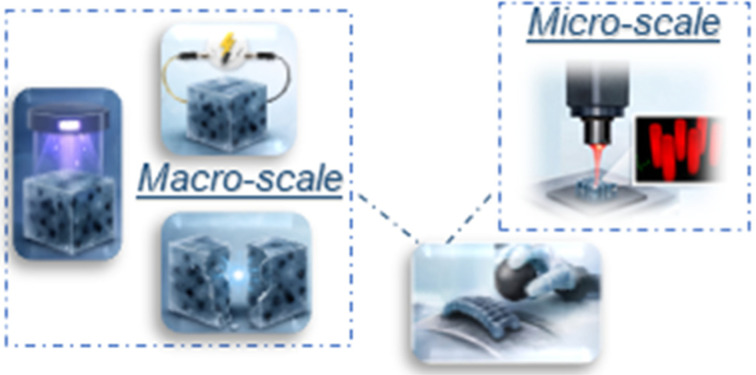

## Introduction

Soft robotics is an emerging field that requires materials with properties similar to those of soft biological matter.^1,2^ Hydrogels are promising materials for soft robotic systems, defined as highly hydrophilic, cross-linked polymeric networks. Their hybrid structure allows them to have characteristics that perfectly resemble the viscoelastic properties of many soft tissues.^1,3,4^ Specifically, the mechanical properties of hydrogels, together with their inherent softness, make them more advantageous for the design and manufacture of soft robotic systems^5^ and fine-tuning their interaction with living systems.^6^

Another notable property of certain hydrogels is their stimulus-responsive behavior, defined as the ability to undergo reversible changes in their properties in response to specific external stimuli such as temperature, pH, electric or magnetic field, light and chemical stimuli.^7–9^ Owing to this stimulus responsiveness, the properties and behavior of these hydrogels can be precisely controlled and modified in specific environments, making them extremely useful materials for many soft robotic applications.^10–13^ Within this class of materials, conductive hydrogels are increasingly explored for soft robotic applications, as they unite electrical conductivity with tissue-like mechanical properties, covering an exceptionally broad modulus window from Pa to MPa.^14–18^ In this context, integration of conductive hydrogels into soft robotic systems could enable more advanced and adaptive functionalities beyond conventional actuation and sensing. Specifically, concepts emerging from next-generation electronic systems, such as autonomous and environment-adaptive electrochemical devices, demonstrate how functional materials can dynamically respond to changing external conditions. Extending such adaptive material concepts to hydrogels highlights their potential to support more resilient, reliable, and intelligent behavior in soft robotic platforms.^19^

However, the successful implementation of electroconductive hydrogels in soft robotics depends on several key factors, including their structural integrity, conductivity, biocompatibility, and durability. Furthermore, achieving precise microstructural control while maintaining bulk mechanical stability remains a significant challenge. Addressing these challenges requires an advanced fabrication approach that allows multiscale structuring of the hydrogel network, ensuring both macroscale robustness and microscale functional precision.

Recent advancements in hydrogel-based soft robotic systems have focused on integrating conductive components to enhance electrical responsiveness and functionality. Conductive polymers, such as polyaniline (PANI), polypyrrole (PPy), and poly(3,4-ethylenedioxythiophene) (PEDOT), have been extensively studied due to their tunable conductivity and biocompatibility.^20–22^ Additionally, conductive particles, particularly metallic nanoparticles, carbon nanotubes and graphene derivatives, have been incorporated into hydrogels to enhance conductivity while maintaining mechanical flexibility.^17,23–26^ However, challenges persist in achieving a homogeneous distribution of these conductive fillers within the hydrogel matrix, ensuring long-term stability, and developing scalable fabrication methods that allow precise patterning at multiple length scales. In this context, the combination of classical macroscale fabrication techniques with advanced microscale structuring approaches presents a promising strategy for developing high-performance electroconductive hydrogels for soft robotics. Additionally, the hybrid strategy involving the incorporation of conductive particles into conjugated conducting polymer matrices has emerged as another effective alternative methodology to fabricate ECHs with tunable and improved properties for targeted applications.^27,28^

Self-healing is also crucial for electroconductive hydrogels employed in soft robotic systems because it enhances their durability, functionality, and long-term performance. Soft robots operate in dynamic environments where mechanical stress, deformation, and minor damage are inevitable. A self-healing hydrogel can autonomously repair cracks or breaks, restoring electrical conductivity and mechanical integrity, thus extending the lifespan of the system without external intervention and improving its reliability in practical applications.^29,30^ This self-healing behavior is enabled by reversible chemical interactions within the polymer network, including non-covalent bonds such as hydrogen bonding, host–guest interactions, and π–π stacking, as well as dynamic covalent bonds such as imines, disulfides, and boronate esters, which allow the network to re-form after disruption.^31^

In this study, we present the fabrication of multifunctional electroconductive hydrogels at both macro- and micro-scales, intended for potential applications in soft robotic systems. The hydrogel network is based on methacrylated gelatin (GelMa), a widely preferred biopolymer known for its tunable mechanical properties, biodegradability, and excellent biocompatibility.^32^ Electrical conductivity was introduced into hydrogels by using a hybrid strategy. Polyaniline–silver nanoparticles (PANI–AgNPs) were synthesized through a one-pot reaction to ensure not only uniform distribution of conductive particles but also stronger interfacial interactions and more cost-effective production with environmental sustainability compared to the two-step process. The fabrication of GelMa/PANI–AgNP hydrogels was carried out at two different scales to demonstrate that these ECHs can be produced at both scales using the same formulation: macroscale fabrication is achieved *via* ultraviolet (UV) crosslinking, while microscale structuring is performed using two-photon polymerization (2PP), ensuring the fabrication of highly intricate three-dimensional microstructures with submicron resolution.^33–35^ This dual-fabrication approach allows for the creation of mechanically stable bulk hydrogel structures while enabling precise control over microscale architecture, which is critical for applications requiring localized electrical conductivity and responsiveness. While the present study does not implement a hybrid or fully integrated multiscale workflow, it demonstrates the feasibility of the same material formulation at both macro- and micro-scales and provides a foundation for future exploration of electroconductive hydrogels. Such independent compatibility at different length scales may be valuable for soft robotic components or smart biomaterials, where bulk properties and microscale features can be tailored according to application requirements.

The fabricated macro-scale GelMa/PANI–AgNP hydrogel was systematically characterized to evaluate its chemical, electrical, mechanical, and biological properties. Electrochemical analysis using cyclic voltammetry and four-point probe measurements confirmed its sufficient conductivity and stability. Mechanical properties, including compressive strength and Young's modulus, were assessed using compression tests, demonstrating the hydrogel's suitability for dynamic soft robotic motion. *In vitro* cytocompatibility studies with rat embryonic fibroblast 52 wild-type (REF52 WT) cells further verified its biocompatibility, supporting its potential for bio-integrated robotic systems.

Moreover, the macro-scale GelMa/PANI–AgNP hydrogel exhibited self-healing behavior, validated through mechanical recovery, conductivity restoration, and CV measurements, effectively repairing physical damage at the macro-scale. Unlike many previously reported conductive hydrogels, the present design integrates PANI–AgNP hybrid components within the GelMa network to form a homogeneous and percolated conductive pathway while simultaneously enabling autonomous self-healing through dynamic non-covalent interactions, including hydrogen bonding, ionic interactions, and π–π stacking. This combination of features ensures both mechanical robustness and electrical recovery, which are rarely achieved concurrently in prior systems.^36,37^ Importantly, the demonstrated approach is compatible with different fabrication scales, from macro-scale bulk hydrogels to micro-fabricated structures, highlighting the versatility and scalability of the design for practical applications. Overall, the results highlight the multifunctional nature of the hydrogel, including adequate mechanical and electrical performance, water absorption, degradability, stability, cytocompatibility, and a reliable self-healing capacity. These findings suggest that the demonstrated macro-scale performance can be translated to micro-fabricated structures, emphasizing the material's versatility. This combination of properties underlines its potential for robust and long-lasting use in soft robotic systems.

## Materials and methods

### Materials

Gelatin (Gel, from bovine skin, Type B, powder, 225 g of bloom), methacrylic anhydride (Ma, *ρ*: 1.035 g ml^−1^), phosphate buffered saline (PBS), silver nitrate (AgNO_3_, ACS reagent, ≥99.0%), aniline (ANI, ACS reagent, ≥99.5%), hydrochloric acid (HCl, ACS reagent, 37%), ethanol (EtOH, absolute, reag. ISO, ≥99.8%), ammonium persulfate (APS, ≥99.5%), Irgacure 2959, (2-hydroxy-4′-(2-hydroxyethoxy)-2-methylpropiophenone), rhodamine B and lithium-phenyl-2,4,6 trimethylbenzoylphosphinate (LAP, ≥95%) were purchased from Sigma Aldrich. 3.5 kDa cut off dialysis membrane tubing was purchased from Spectra/Por®. Cell culture chemicals and solutions including Dulbecco's Modified Eagle Medium (DMEM), fetal bovine serum (FBS), and antibiotic (penicillin/streptomycin) were purchased from PAN Biotech (Germany). The WST-8 assay kit was provided by Sigma Aldrich. All chemicals were used as supplied without further purification.

### Synthesis of methacrylated gelatin (GelMa)

GelMa was synthesized *via* methacrylation carried out by the substitution reaction between the amino and hydroxyl functional groups in the side chains of Gel and the methacryloyl group coming from Ma. Briefly, 20 g of Gel was dissolved in 200 mL of PBS at 50 °C for 1 h. Following that, 16 mL of Ma was slowly added to the Gel solution at 50 °C and stirred for 3 hours. After dilution with an additional 400 mL of PBS, the obtained solution was dialyzed against deionized water at 40 °C for 7 days in order to remove unreacted Ma and salts from the diluted solution. The purified final mixture was lyophilized to obtain GelMa in the form of white foam and stored at −20 °C for further use.

### One-pot synthesis of polyaniline–silver nanoparticles (PANI–AgNPs)

A one-step reaction mechanism was used to synthesize PANI–AgNPs. Firstly, 0.2 M aniline monomer and 0.44 mmol of AgNO_3_ were dissolved in 1.0 M HCl. Then, 0.04 M APS solution was separately prepared in 1.0 M HCl as an oxidizing agent. The prepared solutions were mixed and kept under static conditions for 12 h at room temperature. The resultant product was separated by centrifugation and thoroughly washed with deionized water and ethanol in sequence. In the final step, dark blue-greenish powders were obtained after drying at 60 °C under vacuum for 24 h.

### Fabrication of macro-scale GelMa/PANI–AgNP hydrogels

10% (w/v) of GelMa solution was prepared by dissolving freeze-dried GelMa samples in PBS at 40 °C under continuous stirring. Subsequently, 0.5% (w/v) Irgacure 2959 was added into the polymer solution as a photoinitiator. In order to fabricate macro-scale GelMa hydrogels, the final mixture was exposed to UV light (365 nm) for 2 min.

On the other hand, a 0.1% (w/v) stock PANI–AgNP solution was prepared in PBS. Different amounts of stock PANI–AgNP solution (2%, 5% and 10% v/v) were added into the initial 10% (w/v) GelMa solution with a 0.5% (w/v) photoinitiator. To fabricate macro-scale GelMa/PANI–AgNP hydrogels, the resulting mixture was exposed to the same conditions employed in the previous step.

### Fabrication of micro-scale GelMa/PANI–AgNP hydrogels by two-photon 3D laser printing

The resist formulation was adapted from Erben *et al.* Briefly, a 25% (w/v) GelMa solution was prepared in PBS at 40 °C. LAP was dissolved in PBS to prepare a stock photoinitiator solution with a concentration of 340 mM. The prepared GelMa solution was mixed with stock PANI–AgNPs and the stock LAP solution with a final concentration of 10% (v/v) of PANI–AgNPs and 68 mM LAP. The obtained photoresist was kept in the dark.

3D micro-scale GelMa/PANI–AgNP hydrogels were fabricated with a commercially available two-photon polymerization (2PP) setup (Photonic Professional GT2, Nanoscribe GmbH & Co. KG) using an oil immersion configuration with a 25×, NA = 0.8 immersion objective. The optimization study for printing parameters was performed in the range of 30–100% laser power scaling factor (relative to the laser power in the back focal plane, *i.e.* 66 mW) and the scanning speed was varied from 30 to 110 mm s^−1^. The laser power scaling factor and the scanning speed were set as 90% and 30 mm s^−1^, respectively, as optimized printing parameters for the micro-scale GelMa/PANI–AgNP hydrogels. They were printed onto silanized slides attached to the sample holder. After printing, the micro-scale hydrogels were developed for 10 min by using 10 ml of DPBS to remove the unpolymerized resin. In the final step, the samples were stored in DPBS at room temperature for further use.

### Characterization

The synthesized GelMa, PANI–AgNPs and fabricated macro-scale hydrogels (GelMa and GelMa/PANI–AgNPs) were characterized using various analytical techniques to determine their structural and morphological properties. First, the chemical structures of the materials were identified using Fourier Transform Infrared Spectroscopy (FT-IR, Jasco FT-IR 4600, Tokyo, Japan). The spectra were recorded in the range of 500–4000 cm^−1^ to confirm the presence of characteristic functional groups.

The methacrylation degree of GelMa was determined using Proton Nuclear Magnetic Resonance Spectroscopy (^1^H-NMR). Gel and GelMa were dissolved in deuterium oxide (D_2_O) (30 mg ml^−1^) and spectra were recorded with a Bruker AVANCE III 600 MHz spectrometer operating at 40 °C. The obtained chemical shifts (*δ*, ppm) were analyzed to confirm the methacrylation reaction, and accordingly, the degree of methacrylation (DoF) was calculated by using the following eqn (1):1
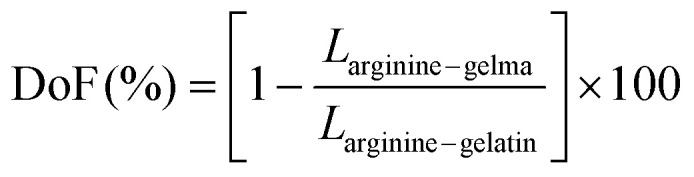
where *L*_arginine–gelma_ and *L*_arginine–gelatin_ are the integration signals of arginine from GelMa and Gel structures, respectively.

To verify the characteristic absorption features of PANI–AgNPs, UV-Vis spectroscopic analysis was carried out by dispersing the conductive particles in MilliQ water. The absorption spectrum was recorded on a Jasco V-770 spectrophotometer within the 200–800 nm region. The hydrodynamic diameter and particle size distribution of PANI–AgNPs dispersed in PBS were investigated using a Dynamic Light Scattering (DLS) system (Zetasizer Ultra/Pro-Malvern Panalytical, Japan). The measurements were performed with a scattering angle of 173° at 25 °C. The morphological characterization of GelMa and macro-scale GelMa/PANI–AgNP hydrogels was performed by using a scanning electron microscope (SEM, JEOL JSM-7610F, Tokyo, Japan). Dry samples were fixed on a substrate and sputter-coated with an 80% gold, 20% palladium alloy using a Leica ACE600 sputter coater. On the other hand, the morphology of the synthesized PANI–AgNPs was examined using a transmission electron microscope (TEM, JEOL JEM-1400) at an acceleration voltage of 80 kV. The particles were diluted in distilled water and deposited on a 300 mesh copper grid filmed with a pioloform matrix for support.

### Swelling test

The swelling behavior of the GelMa and GelMa/PANI–AgNP hydrogels was determined by immersing the samples in PBS (pH 7.4) at 37 °C. Initially, the prepared hydrogel specimens were weighed in the dry state (*W*_dry_) and then incubated in PBS medium. At different time intervals, the swollen samples were removed from the medium, gently blotted with filter paper to remove excess surface water, and immediately weighed (*W*_swollen_). The swelling ratio (SR) of the hydrogels was calculated using the following eqn (2):2
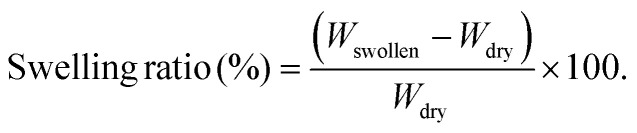


### Hydrolytic degradation

The hydrolytic degradation test of the GelMa and GelMa/PANI–AgNP hydrogels was carried out by immersing pre-weighed samples in 10 ml of PBS medium at 37 °C. At determined time intervals (1, 7, 14, and 21 days), samples were carefully removed from the solution, and after surface wiping, they were dried in a vacuum oven at 40 °C until a constant weight was reached. The following equation (eqn (3)) was used for the calculation of sample mass loss:3
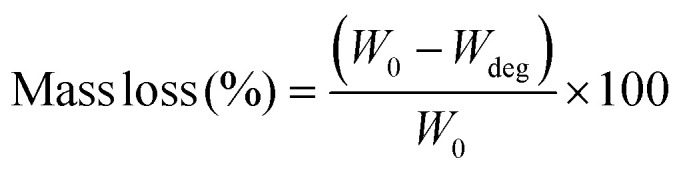
where *W*_0_ is the original dry mass and *W*_deg_ is the remaining dry mass after degradation.

### Mechanical performance

The mechanical properties of the GelMa and GelMa/PANI–AgNP hydrogels under compression were evaluated by using a Zwick/Roell universal testing machine equipped with a 5 N load cell (2.5 kN Zwicki, Ulm, Germany). The prepared hydrogels with a height of 6 mm and a diameter of 4.5 mm were tested at room temperature with a pre-load of 0.1 N and a cross-head speed of 10 mm min^−1^. The Young's modulus was calculated from the slope of the linear region of the stress–strain curve. Additionally, the ultimate compressive strength, defined as the maximum stress before failure, was determined to evaluate the hydrogel's ability to withstand mechanical deformation.

### Electrochemical characterization

The electrical conductivity of the fabricated hydrogels was determined using the four-point probe technique with a Keithley 2450 source meter (Tektronix, USA) as a digital multimeter. Prior to electrical measurements, the hydrogel samples were removed from PBS and gently dried to eliminate excess surface PBS. All conductivity measurements were conducted in the absence of any external liquid medium. The four probes were placed in direct contact with the hydrogel surface, and a constant voltage was applied while the corresponding current was measured. The sheet resistance (*ρ*) was calculated using the following eqn (4):

4 *ρ* = 2π*Rs*

where *s* is the probe spacing (cm) and *R* is the measured resistance value (Ω).

The electrical conductivity of the hydrogels (*σ*) was then determined using eqn (5):5
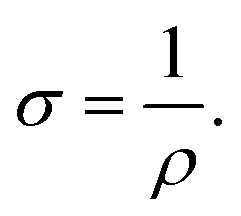


To qualitatively evaluate the electrical conductivity of the GelMa/PANI–AgNP hydrogel, a light bulb experiment was conducted. A simple electrical circuit was assembled, consisting of a DC power source (Keithley 2450 source meter), a small LED and a conductive hydrogel sample as the connecting element. When the power source was activated, the brightness of the LED was observed as an indicator of the hydrogel's ability to conduct electricity.

The electrochemical behavior of the prepared GelMa/PANI–AgNP hydrogels was analyzed *via* CV measurements performed by using an Autolab PGSTAT204 potentiostat/galvanostat with NOVA 2.0 software (Metrohm Autolab). A custom-made sandwich-type electrode system was used, in which the hydrogel was placed between two electrodes to ensure stable electrical contact. The CV measurements were carried out at room temperature, with a potential window of −1 V to 1 V and a scan rate of 100 mV s^−1^. The obtained CV curves were used to evaluate the electrochemical behavior of the fabricated GelMa/PANI–AgNP hydrogels considering their long-term stability, reproducibility, storage stability characteristics and redox behavior.

### Self-healing and adhesive properties

The self-healing ability of the fabricated GelMa/PANI–AgNP hydrogel was evaluated through both qualitative and quantitative methods. To visually observe self-healing, the hydrogel sample was first cut in the middle and then the two parts of the hydrogel were merged together from the fracture surfaces by slightly pressing at room temperature. For quantitative analysis, both original and healed GelMa/PANI–AgNP hydrogel samples were then subjected to mechanical and electrical characterization tests to determine the self-healing efficiency (*η*) calculated by using eqn (6):6
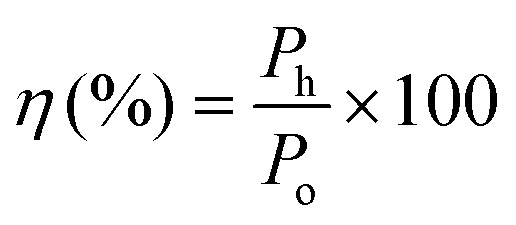
where *P*_h_ is the healed property measured after healing and *P*_o_ is the original property measured before healing.

The adhesive characteristic of the GelMa/PANI–AgNP hydrogel was qualitatively examined by visual observation of its attachment to the surfaces of different materials.

### Cytotoxicity test

Rat embryonic fibroblast cells (REF52 WT) and the WST-8 reduction assay (Sigma Aldrich) were used to evaluate the cell viability of the GelMa and GelMa/PANI–AgNP hydrogels. The assay was performed in agreement with ISO-10993.

REF52 cells were cultured in Dulbecco's modified Eagle's medium (DMEM) supplemented with 10% fetal bovine serum (FBS, PAN Biotech) and 1% penicillin/streptomycin at 37 °C and 5% CO_2_. At passage 10, *ca.* 5000 rat embryonic fibroblast cells were seeded into a 96-well microplate and incubated in 100 μL of cell culture medium for 24 h. Afterwards, the GelMa and GelMa/PANI–AgNP hydrogels were incorporated into the 96-well microplate with REF52 cells; prior to this, they were sterilized under UV light for 30 minutes and washed with PBS. The hydrogels were incubated with REF52 cells for another 24 h. After removing the hydrogels, REF52 cells were washed with PBS to later add 10 μL of WST-8 solution to 100 μL of cell media. Following 4 h of incubation, 50 μL of the supernatant of each sample was measured at 450 nm with a spectrometer (Epoch 2, BioTek). For the analysis of the data, the background value, which is the absorbance of the cell medium with the 10 μL of WTS-8, was subtracted from all the obtained values, and the absorbance values were normalized to the absorbance of the controls.

### Optical imaging of micro-scale GelMa/PANI–AgNP hydrogels

Optical imaging including autofluorescence and two-photon imaging was employed to capture high-resolution images of the microstructures fabricated using a two-photon polymerization process.

#### Autofluorescence imaging

All image stacks were performed using a FLUOVIEW™ FV4000 confocal laser scanning microscope (Evident Europe GmbH, formerly Olympus Scientific Solutions, Tokyo, Japan), equipped with a SilVIR™ silicon photomultiplier (SiPM) detector featuring patented fast signal processing technology. A 20× universal plan fluorite objective lens (UCPLFLN20X, NA 0.7; Olympus Corporation, Tokyo, Japan) was used for image acquisition. Autofluorescence was excited with a 488 nm laser and a 561 nm laser (Coherent OBIS LS, Coherent Inc., Santa Clara, CA, USA), with emission collected between 500 and 540 nm and 580 and 620 nm, respectively. The acquired data were organized and visualized using the native FLUOVIEW software suite (Evident Europe GmbH).

#### Two-photon imaging

Two-photon fluorescence imaging was carried out using the same laser scanning microscope integrated with a Spectra-Physics InSight® X3 Dual Wavelength Femtosecond Laser (MKS Instruments, Andover, MA, USA). Excitation was achieved at 1045 nm, and emitted fluorescence was captured in the 580–620 nm spectral range *via* the microscope's non-descanned detection (NDD) system.

The imaging was conducted under ambient conditions, and the hydrogel samples were submerged in PBS buffer to avoid dehydration and to maintain their native state during imaging.

### Statistical analysis

All experimental data were analyzed by using OriginPro 2018 software, and results are expressed as the means of three separate experiments (*n* = 3) with standard deviation.

## Results and discussion

**Fig. 1:**
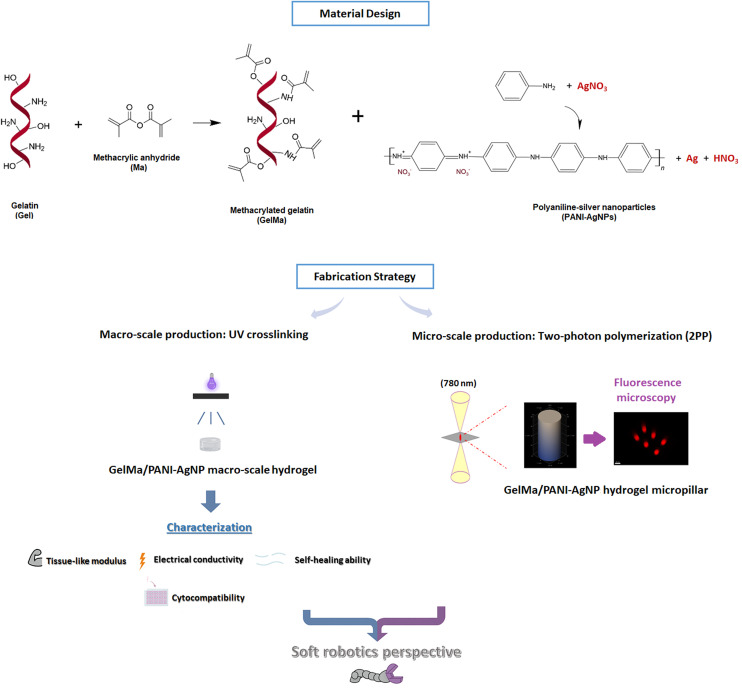
Overview of GelMa/PANI–AgNP hydrogel fabrication using complementary macro- and microscale approaches, including UV-crosslinked bulk structures and 2PP-fabricated microscale 3D architectures, with key mechanical, electrical, cytocompatibility, and self-healing assessments.

Fig. 1 presents a conceptual scheme summarizing the main steps of the study, including materials synthesis, hydrogel fabrication at different length scales, and characterization of the developed functional materials. This figure aims to offer a clear overview of the workflow and highlight the interrelation between the different stages discussed in the following sections.

### Synthesis and characterization of GelMa

**Fig. 2:**
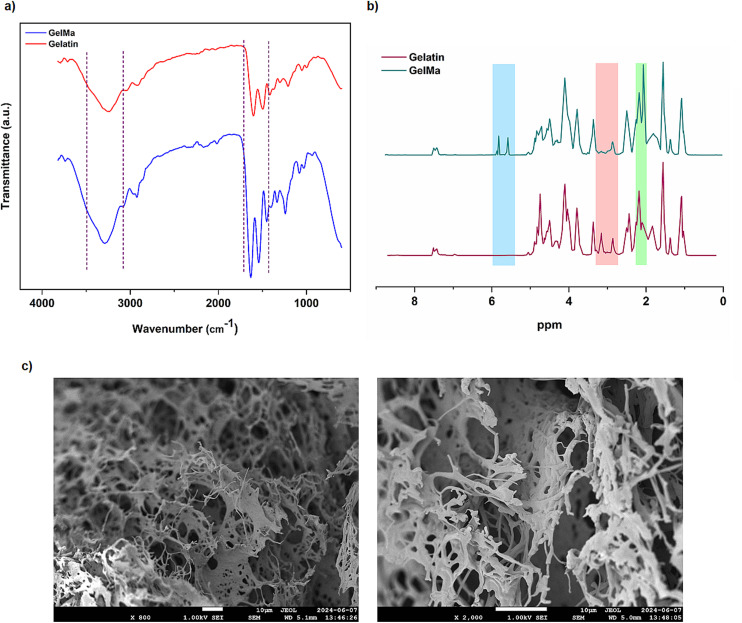
(a) FT-IR spectra of Gel and GelMa. (b) ^1^H-NMR spectra of Gel and GelMa. (c) Representative SEM images of the synthesized GelMa obtained from the same sample at different magnifications, showing (left) the overall microstructure and (right) a higher-magnification view revealing finer features of the GelMa polymer network. The freeze-dried GelMa sample was mounted on an aluminum stub, sputter-coated with an 80 : 20 gold–palladium alloy, and imaged under high vacuum. The SEM images demonstrate the porosity of the material.

To confirm the synthesis of GelMa, the pure gelatin and synthesized GelMa were chemically characterized by using FTIR analysis (Fig. 2a). The characteristic peaks of Gel attributed to the stretching vibration of N–H and O–H bonds at 3300 cm^−1^ and the C–H stretching vibration at 2900 cm^−1^ were observed in the FTIR spectra of both Gel and GelMa.^38^ In addition to these characteristic peaks, noticeable changes in relative peak features appeared at about 1147, 1543 and 1625 cm^−1^ corresponding to C–O, C=C, and C=O stretching vibrations, respectively, which proved the successful insertion of methacrylate groups into the Gel structure.^39^ The intensity of the peak in the 3200–3400 cm^−1^ range increased after methacrylation, which revealed the presence of peptide bonds (N–H stretching). This could be considered as another indication of the methacrylation process. Additionally, a slight shift (∼40 cm^−1^) observed in certain bands is attributed to changes in the local chemical environment and intermolecular interactions following methacrylation.^40^

The methacrylation degree of GelMa was determined using ^1^H-NMR analysis. Methacrylate functional groups were grafted onto the Gel backbone *via* the reaction between methacrylic anhydride and arginine units. Accordingly, the modification of the gelatin structure with methacrylate groups was confirmed by the decrease in the arginine signal at 2.8–3.1 ppm and also the increase in the methyl group signal at 2.0–2.2 ppm in the GelMa spectrum (Fig. 2b). Compared to the spectrum of Gel, the new peaks observed at 5.6–5.8 ppm in the ^1^H-NMR spectrum of GelMa correspond to the acrylic protons of methacrylic functions, which is another confirmation of the methacrylation.^38,41^ The degree of functionalization was calculated to be 78% based on the ratio of the integrated areas of the amine groups of GelMa and Gel.

SEM analysis was performed to examine the morphological structure of the synthesized GelMa. The SEM images with different magnifications (Fig. 2c) clearly showed the characteristic thread-like porous structure of GelMa. The observed interconnected porosity presumably arises from phase separation processes during drying, where polymer–solvent interactions and rapid solvent removal create micro-voids within the bulk material.

### Synthesis and characterization of PANI–AgNPs

**Fig. 3:**
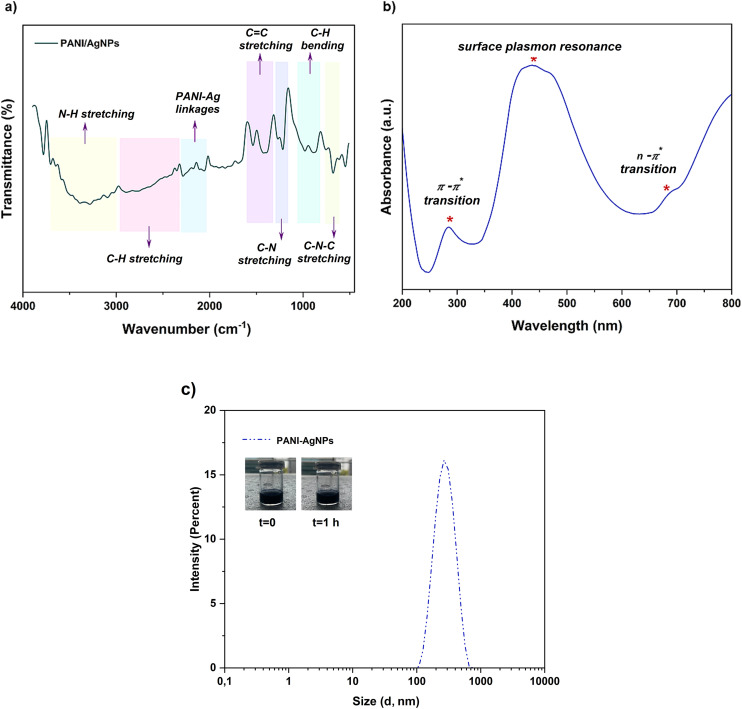
Characterization of the synthesized PANI–AgNPs *via* a modified one-pot reaction mechanism. (a) FT-IR spectrum of PANI–AgNPs. (b) UV-Vis spectrum of PANI–AgNPs. (c) Particle size distribution of PANI–AgNPs. The DLS profile of the PANI–AgNPs shows a narrow size distribution with acceptable homogeneity (PDI < 0.3) and an average hydrodynamic diameter of 270 nm.

The chemical structures of the produced PANI–AgNP conductive particles were characterized using both FT-IR (Fig. 3a) and UV-Vis analyses (Fig. 3b). The characteristic peaks and absorbance values arising from both PANI and Ag structures confirmed the successful formation of the PANI matrix with embedded Ag nanoparticles. As shown in the FT-IR spectrum of PANI–AgNPs, the broad peak in the 3500–3000 cm^−1^ region and that in the 2900–2300 cm^−1^ region corresponded to the N–H bonding of the substituted amides and the stretching vibrations of C–H peaks, respectively. The bands observed at approximately 1532 cm^−1^ and 1425 cm^−1^ corresponded to the C=C stretching vibrations of the quinoid and benzenoid rings, respectively, which is a clear indication of the emeraldine base form of PANI.^42,43^

The C–N stretching vibration of the secondary aromatic amine appeared at around 1250 cm^−1^ and the C–H out of plane bending was observed near 1050 cm^−1^ and 850 cm^−1^, providing further confirmation of the conductive form of PANI. In addition to these, the absorption band observed at around 680 cm^−1^ corresponded to C–N–C stretching vibration in the structure.^44,45^

The UV-Vis absorption spectra of PANI–AgNPs exhibited two absorption bands: one at around 285 nm, attributed to the π–π* transition of the benzenoid rings, and the other at 683 nm, corresponding to the n–π* transition of the quinoid structure. A distinct surface plasmon resonance peak was observed at 436 nm, which is a typical feature of AgNPs.^46,47^ This peak confirms the formation of AgNPs during the polymerization reaction of aniline using AgNO_3_. The intensity and slight broadening of this peak suggest good dispersion of AgNPs and possible interactions between the nanoparticles and the PANI chains. The obtained UV-Vis results also indicated the strong interfacial interaction between the two components.

DLS analysis was conducted to determine the hydrodynamic diameter and particle size distribution of the synthesized PANI–AgNP composite. The results (Fig. 3c) revealed an average particle size of approximately 270 nm, indicating the successful formation of nanoscale composite structures. The polydispersity index (PDI) was found to be 0.27, suggesting a moderately narrow size distribution. Typically, a PDI value below 0.3 indicates an acceptable uniformity in nanoparticle size, suitable for various nanomaterial applications.^48^ These findings support the successful synthesis of a mostly monodisperse PANI–AgNP composite and also correlate well with UV-Vis results, further confirming the stable formation and dispersion of the composite in colloidal form.

TEM analysis was employed to investigate the morphological properties of the synthesized PANI–AgNP composite and the corresponding micrographs are provided in Fig. S1 (SI).

### Characterization of macro-scale GelMa/PANI–AgNP hydrogels

**Fig. 4:**
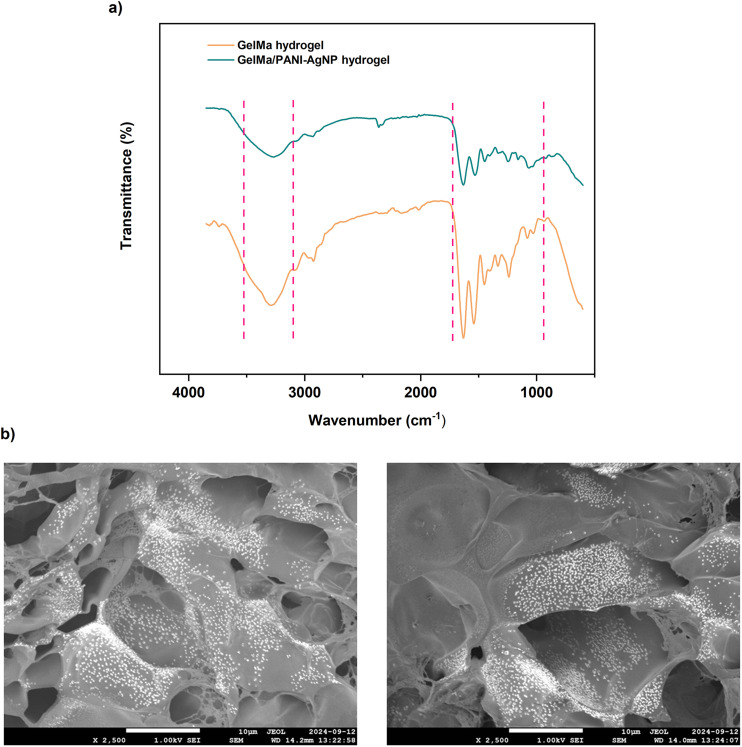
Chemical and morphological characterization of macro-scale hydrogels. (a) FT-IR spectra of the fabricated pure GelMa and GelMa/PANI–AgNP hydrogels. (b) Cross-sectional SEM images of air-dried hydrogel samples with a novel ternary composition, acquired at the same magnification from different regions, illustrating the successful integration and distribution of conductive PANI–AgNPs within the GelMa polymeric network.

In order to investigate the successful integration of PANI–AgNPs into the GelMa network, FT-IR analysis was performed for both pure GelMa and GelMa/PANI–AgNP hydrogels (Fig. 4a). In both spectra, characteristic peaks of the GelMa structure attributed to C=O stretching, N–H bending coupled with C–N stretching, O–H and N–H stretching vibrations were observed. Upon incorporation of PANI–AgNPs into the hydrogel network, most of these characteristic peaks were retained in the FT-IR spectrum of the GelMa/PANI–AgNP hydrogel, since many of the characteristic peaks of the PANI–AgNP structure lie within wavenumber regions similar to those of the native GelMa hydrogel.^49^ As a result, the primary differences between the pure GelMa and the GelMa/PANI–AgNP hydrogel spectra were observed as changes in peak intensities and subtle band shifts due to intermolecular interactions between GelMa and the PANI–AgNPs, possibly *via* hydrogen bonding or electrostatic interactions. This observation supports that PANI–AgNPs are well embedded in the GelMa network without disrupting the core polymer chemical bonds.

SEM was utilized to investigate the morphological features of GelMa/PANI–AgNP hydrogels. The obtained SEM images (Fig. 4b) revealed a porous architecture typical of hydrogel networks, with clearly visible interconnected pores distributed throughout the matrix. The particles were observed as distributed, nano-scale clusters embedded within some regions of the hydrogel matrix. These features indicate that the PANI–AgNPs were successfully integrated into the GelMa structure without disrupting the porous architecture required for biological interaction and flexibility. The distribution of PANI–AgNPs throughout the hydrogel also suggests good compatibility between the GelMa and PANI–AgNP phases, which is crucial for ensuring consistent electrical performance across the hydrogel scaffold.

### Swelling behavior

**Fig. 5:**
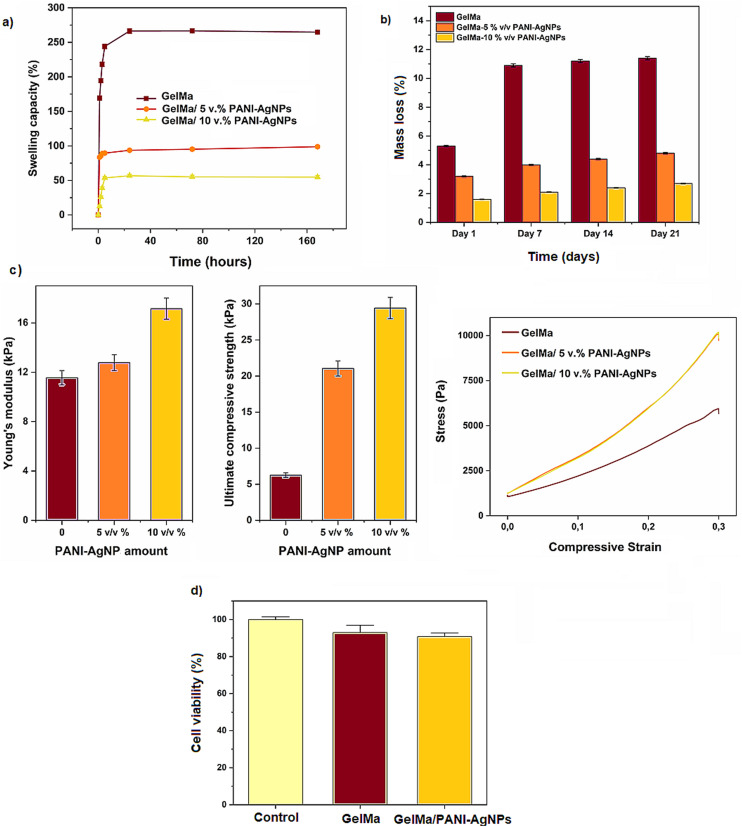
(a) Swelling behavior of hydrogels with different amounts of PANI–AgNPs. (b) Hydrolytic degradation profiles of hydrogels with different amounts of PANI–AgNPs. (c) Mechanical performance of the produced hydrogels regarding Young's moduli, ultimate compressive strengths and stress–strain curves of the produced hydrogels under compression at room temperature. The results confirm the combination of elasticity and strength with soft, tissue-like behavior for the fabricated materials. (d) Cell viability results of rat embryonic fibroblast cells (REF52 WT) in contact with GelMa and GelMa/PANI–AgNP hydrogels with a WST-8 assay. They show high viability of the produced hydrogels. Error bars denote standard deviation.

The swelling behavior of the hydrogels was evaluated with different conductive component concentrations (Fig. 5a). Pure GelMa hydrogels exhibited the highest swelling ratio among all samples tested. This result can be attributed to the highly porous and hydrophilic nature of the GelMa matrix, which allows for significant water uptake capacity due to the presence of abundant functional groups that facilitate hydrogen bonding with water molecules.

On the other hand, the incorporation of PANI–AgNPs into the GelMa matrix led to a noticeable decrease in the swelling ratio. Compared to GelMa, the more hydrophobic nature of PANI reduced the hydrophilicity of the conductive hydrogels.^50^

It could also be clearly seen that as the concentration of PANI–AgNPs increased, the degree of swelling further decreased. This behavior can be explained by the reduction of porosity in the hydrogel structure and also the formation of a denser structure. The conductive particles may partially fill the pores in the hydrogel structure and prevent the formation of the interconnected porous network. As a consequence, this can lead to limited space for water molecules. Furthermore, the incorporation of PANI–AgNPs into the polymeric network contributed to a denser polymer network because of the additional crosslinking points or physical entanglements, which resulted in the restriction of the water absorption ability of the produced hydrogel. Although the incorporation of PANI–AgNPs into the pure GelMa hydrogel structure led to a reduction in swelling, all fabricated hydrogels maintained desirable swelling capacities, ensuring their functional relevance in aqueous and physiological environments.

### Hydrolytic degradation

The hydrolytic degradation behavior of the prepared hydrogels was investigated to evaluate their stability and interaction with aqueous environments under physiological conditions (Fig. 5b). Although GelMa hydrogels are formed *via* UV-induced crosslinking of methacrylate groups, the polymer network still contains hydrolytically labile ester bonds originating from the gelatin backbone and methacrylate modification. Upon exposure to an aqueous environment, water molecules diffuse into the hydrogel matrix and initiate the hydrolysis of these ester bonds, leading to network cleavage and mass loss.^51^ As discussed, the pure GelMa hydrogel exhibited the highest swelling ratio, owing to its highly porous and hydrophilic network structure. Consistently, it also demonstrated the highest hydrolytic degradation, with the highest percentage of mass loss observed during the incubation period. This behavior can be attributed to the highly hydrophilic and less cross-linked nature of GelMa, which facilitates greater water uptake and thus accelerates hydrolytic cleavage of bonds within the polymer network.^52^

In contrast, the incorporation of PANI–AgNPs into the GelMa matrix led to a reduction in both swelling capacity and the degradation rate. Specifically, as the concentration of PANI–Ag nanoparticles increased, the percentage of mass loss due to hydrolytic degradation decreased. The positive charges of the ionic polymeric nanoparticles interact with the water and with the hydrogel, disrupting the swelling balance between the GelMa hydrogel and the solvent molecules, reducing the swelling capacity of the material.^53^ Additionally, the interactions of the positive charges of PANI–Ag nanoparticles with the hydrogel contribute to the stability of the structure, diminishing the interactions of the hydrogel with the water molecules and, with it, the hydrolytic cleavage.^54–57^

All hydrogel samples showed a time-dependent increase in mass loss, confirming their partial, time-dependent hydrolytic degradability. However, it could be clearly stated that the rate and extent of degradation were modulated by the presence and concentration of PANI–AgNPs. Overall, the results highlight a clear correlation between reduced swelling behavior and improved hydrolytic stability in the composite hydrogels. While pure GelMa offers high water absorption and faster degradation, the addition of PANI–AgNPs allows for the tuning of degradation rates, offering advantages for applications that require longer-term structural integrity.

### Mechanical characterization

In soft robotic systems, materials are expected to exhibit a unique combination of flexibility, durability, and mechanical compliance to effectively mimic the adaptive, deformable nature of biological tissues. Accordingly, a compression test was performed and the compressive stress–strain curves were obtained to evaluate the mechanical properties including the Young's modulus and ultimate compression strength of both GelMa and GelMa/PANI–AgNP hydrogels with different conductive component concentrations (Fig. 5c). GelMa hydrogels exhibited the lowest Young's modulus of approximately 11 kPa and also the lowest compressive strength value. However, GelMa/PANI–AgNP hydrogels showed higher modulus and compressive strength values compared to the pure hydrogel. The highest values were observed in the GelMa/PANI–AgNP hydrogel with 10% PANI–AgNP content owing to the increased synergistic reinforcing effect of both conductive components.

This increase in mechanical strength could also be attributed to the denser polymer network and physical reinforcement provided by PANI–AgNPs. While GelMa offers excellent biocompatibility and processability, its relatively soft structure limits its functionality in load-bearing or actuation contexts. Conversely, the conductive hydrogels strike a better balance between elasticity and robustness, key for soft robotic systems that require repeated deformation and mechanical resilience.

In the compressive stress–strain curves of the developed hydrogel samples, all formulations displayed the typical non-linear response of cross-linked hydrogel networks.^58^ The curve of the GelMa/10 v% PANI–AgNP hydrogel reached the highest compressive stress, indicating a more robust load-bearing network compared to the pure GelMa hydrogel. The steeper slope and the larger area under the curve observed for the GelMa/10 v% PANI–AgNP formulation reflect enhanced network integrity and a higher capacity for mechanical energy dissipation under compression, whereas the gentler slope of the curve for the GelMa hydrogel reflects a more compliant and deformable matrix. This behavior can be attributed to the reinforcing effect of conductive PANI–AgNPs, which restrict polymer chain mobility and stabilize the porous structure during deformation. Overall, variations in conductive particle content resulted in distinct viscoelastic responses, with the GelMa/10 v% PANI–AgNP hydrogel demonstrating both the highest energy dissipation capacity and superior recoverability under compressive loading.

Moreover, the modulus ranges of the produced hydrogels (∼10–20 kPa range) fall within or near those of native soft tissues such as muscle, heart and some parts of skin, making them suitable candidates for biomedical interfaces.^59^ Therefore, in the context of soft robotics, these mechanical properties are particularly advantageous. The moderate stiffness and elasticity of these hydrogels make them promising for use in artificial muscles, soft actuators, or biosensing components, where mechanical compliance and durability are essential.

### Cytotoxicity test

The cytotoxicity of the hydrogels was evaluated using the WST-8 cell viability assay. Compared to the control group, where rat embryonic fibroblast cells were grown under standard cultivation conditions, the cell viability values for GelMa and GelMa/PANI–AgNP hydrogels were found to be 92% and 89%, respectively (Fig. 5d), which are comparable to those of previously reported GelMa-based systems.^60^ These results indicate that both hydrogel formulations maintained high cell compatibility, which means that both GelMa and PANI–AgNP components synthesized in this work do not have a negative effect on cell viability.

### Electrochemical characterization

**Fig. 6:**
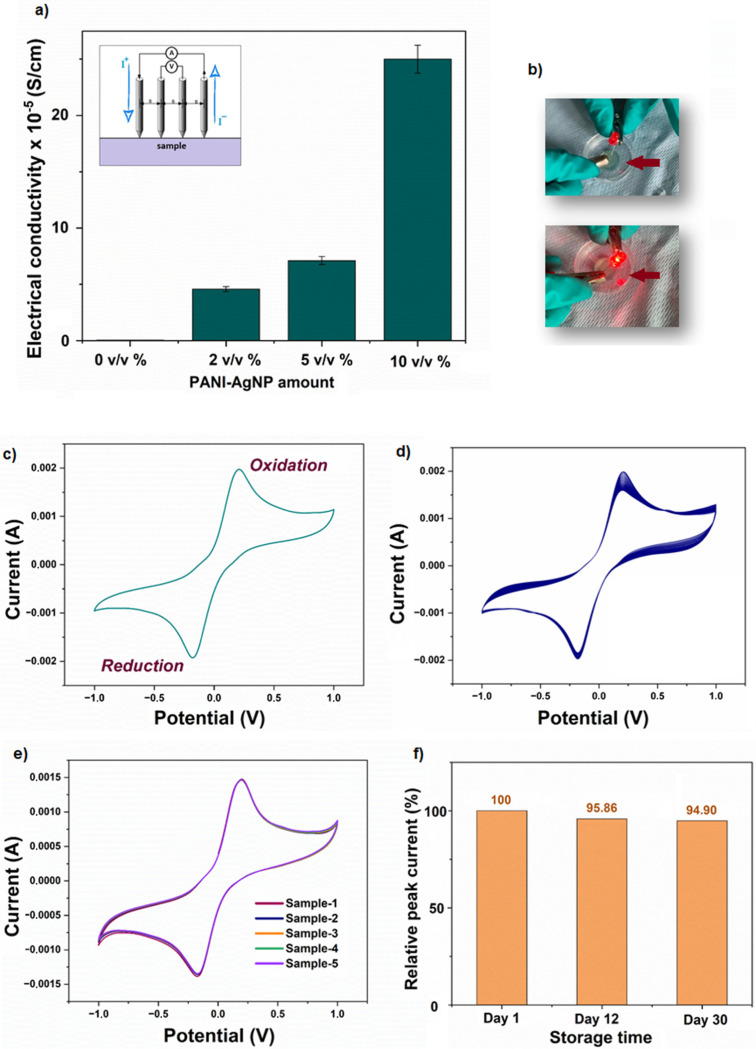
Electrochemical characterization of GelMa/PANI–AgNP hydrogels. (a) Electrical conductivity of GelMa/PANI–AgNP hydrogels. (b) Demonstration of electrical conductivity through an LED lamp. Photograph of the GelMa/PANI–AgNP hydrogel surface turning an LED light ON. (c) CV response of the GelMa/PANI–AgNP hydrogel, confirming the redox behavior of the fabricated network. (d) Long-term stability test of the GelMa/PANI–AgNP hydrogel over 50 cycles. (e) CV curves of five separate samples of the GelMa/PANI–AgNP hydrogel fabricated using the same formulation and synthesis protocol. (f) Storage stability performance of the GelMa/PANI–AgNP hydrogel for 30 days. (All CV measurements were performed within a potential range of −1.0 to 1.0 V and at a scan rate of 100 mV s^−1^.)

The electrical conductivity properties of the produced hydrogels were proved both using a light bulb experiment, which is a physical method, and using the 4-point-probe method. For the 4-point-probe method, hydrogels with different concentrations and ratios of PANI–AgNPs were tested. The experiments were carried out repeatedly for each sample, taking measurements at three different parts on the sample to analyze the homogeneity of conductivity within the hydrogel network. The obtained consistent results confirmed the homogeneity of the electrical conductivity for the samples. The obtained conductivity values revealed a positive correlation between the concentration of PANI–AgNPs and the electrical conductivity of the hydrogels (Fig. 6a). As the concentration increased, the formation of more continuous conductive pathways within the hydrogel matrix led to improved conductivity, supporting the feasibility of these materials for electro-responsive functions such as sensing or actuation. However, at higher concentrations, particularly above 10% (v/v), significant agglomeration of conductive particles was observed. This aggregation disrupted the uniform distribution of the conductive phase, resulting in inconsistent and non-reproducible conductivity values. Such heterogeneity can impair the performance and reliability of soft robotic components that rely on precise and stable electrical properties. This observation can also be interpreted within the framework of percolation theory, where particle agglomeration near or above the percolation threshold hampers the establishment of a uniform conductive network, thereby preventing the expected improvement in electrical conductivity despite relatively high filler concentrations.^61^ Therefore, 10% (v/v) was determined to be the optimal concentration, balancing enhanced conductivity with structural uniformity and processability. All subsequent electrochemical characterization studies were conducted using this formulation.

In the light bulb experiment, a simple circuit was prepared and a power source was used to test whether the hydrogels were conductive. As can be seen in Fig. 6b, the light bulb is turned ON after a voltage is applied, which proves that the hydrogels acting as circuit elements provide electrical conductivity.

The electrochemical behavior of the conductive GelMa/PANI–AgNP hydrogel was systematically evaluated using cyclic voltammetry (CV) to investigate its redox activity, reproducibility, long-term electrochemical stability, and storage stability, which are the key parameters for its potential use in soft robotic systems.

The CV profile (Fig. 6c) revealed distinct and well-defined oxidation and reduction peaks, indicating a reversible redox process within the hydrogel matrix. This reversible redox behavior in the CV measurements is closely linked to the intrinsic redox transitions of PANI, which is known for its electroactive nature.^62,63^ PANI can exist in three distinct oxidation states: leucoemeraldine (fully reduced), emeraldine (partially oxidized), and pernigraniline (fully oxidized). During the anodic scan, PANI undergoes oxidation from the leucoemeraldine to the emeraldine form, and, with further potential increase, transitions toward the pernigraniline state. These processes are electrochemically reversible, and during the cathodic scan, PANI is reduced back to its original state.

In addition to the intrinsic electroactivity of PANI, the presence of AgNPs within the hydrogel matrix plays a significant supportive role in enhancing the overall redox performance. Due to their excellent electrical conductivity, AgNPs act as efficient electron carriers, facilitating faster and more uniform charge transfer throughout the hydrogel network.^64^ This results in more defined redox peaks and improved electrochemical responsiveness during CV. AgNPs also contribute to the formation of a more continuous and interconnected conductive network by bridging gaps between PANI chains. This synergistic effect promotes stability and repeatability in the redox transitions of PANI. The increased electroactive surface area provided by the AgNPs may also contribute to higher peak currents observed in the CV measurements.

Although silver itself can undergo redox transitions, the dominant electrochemical signals observed in this system are primarily attributed to PANI.^65^ Nevertheless, the presence of AgNPs significantly amplifies the efficiency and reliability of the redox processes, making the hydrogel better suited for applications such as soft robotic actuators or biosensors, where stable and responsive electrochemical behavior is essential.

The fabricated GelMa/PANI–AgNP hydrogel also showed remarkable long-term electrochemical stability (Fig. S2). In a 50-cycle continuous CV test, the redox peak currents and overall curve shapes remained virtually unchanged, indicating minimal structural breakdown under repeated electrochemical stimulation (Fig. 6d). This resilience is particularly advantageous for soft actuators and biosensors, which are frequently exposed to cyclic operation.

The reproducibility of the GelMa/PANI–AgNP hydrogel was confirmed by performing CV measurements on five independently prepared samples (Fig. 6e). The resulting voltammograms exhibited nearly identical responses across all replicates, demonstrating excellent fabrication consistency and homogeneity in conductive network formation. Such reproducibility is essential for practical integration in soft robotic systems, where predictable electrical performance is critical for control and sensing reliability.

Furthermore, the storage stability of the GelMa/PANI–AgNP hydrogel was evaluated by comparing the CV response of hydrogels stored under ambient conditions for 30 days (Fig. 6f). After this period, the relative peak current remained at approximately 95% of its original value. This minimal loss suggests that the fabricated hydrogel maintains its electrochemical integrity over time, supporting its suitability for applications where shelf-life and long-term reliability are essential.

All of these findings confirm that the GelMa/PANI–AgNP hydrogel exhibited reversible redox behavior, high reproducibility, and excellent electrochemical stability, which are highly desirable for soft robotic applications such as artificial muscles, flexible biosensors, and electrically stimulated drug delivery systems. Its ability to maintain functionality during repeated electrochemical cycling and prolonged storage stability performance highlights its potential as a multifunctional material in soft robotic systems.

### Self-healing and adhesive properties

**Fig. 7:**
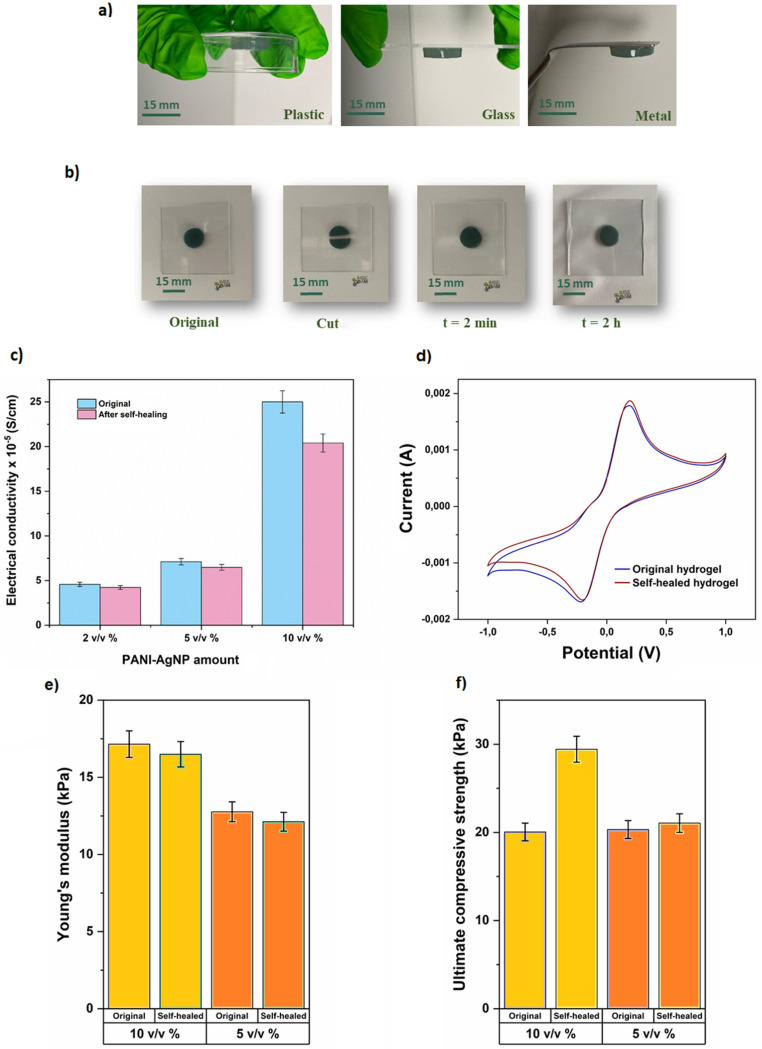
(a) Illustration of adhesive properties of GelMa/PANI–AgNP hydrogels on various material surfaces. (b) Visual demonstration of the self-healing ability arising from the GelMa/PANI–AgNP hydrogel over time, attributed to reversible non-covalent interactions in the polymer network. (c) Electrical conductivity, (d) CV response, (e) Young's modulus, and (f) compressive strength of the GelMa/PANI–AgNP hydrogels before and after self-healing, demonstrating the changes in electrical and mechanical properties induced by the self-healing process. Error bars denote standard deviation.

The adhesion performance of the GelMa/PANI–AgNP hydrogels was qualitatively evaluated by placing the fabricated hydrogels on various substrates, including plastic, glass, and metal surfaces (Fig. 7a). In each case, the hydrogels exhibited good adhesion behavior, maintaining firm contact with the substrates even when turned upside down. No detachment was observed, which indicates sufficient interfacial binding strength. Additionally, observations during sample handling revealed noticeable surface tackiness, further supporting the inherent adhesive character of the hydrated and viscoelastic network.

This adhesive capability is mainly attributed to the chemical structure of the GelMa backbone, which contains abundant polar groups capable of forming hydrogen bonds and van der Waals interactions with different surfaces. The incorporation of PANI chains may support adhesion through hydrophobic interactions and π–π stacking with aromatic or metallic surfaces, enabling stable attachment without additional adhesives for biointerfaces, wearable electronics, or actuator applications.^66^

The self-healing capacity of the GelMa/PANI–AgNP hydrogels was evaluated through both visual observation (Fig. 7b) and functional performance tests, including electrical (Fig. 7c and d) and mechanical characterization (Fig. 7e and f), before and after the damage–repair cycle. Physically, all hydrogel samples exhibited excellent self-healing ability upon simple re-contacting of the cut surfaces without any external stimuli. The cut sections rejoined within minutes under ambient conditions, forming a continuous structure capable of being handled without visible fracture at the healed interface. This behavior is primarily attributed to the dynamic non-covalent interactions within the polymeric matrix.^67,68^ These include hydrogen bonding among Gel chains, reversible physical entanglements, and potential π–π interactions between PANI chains and AgNPs (Fig. S3). Such reversible bonds allow the hydrogel network to autonomously restore its integrity after mechanical disruption, even in the absence of external stimuli, enabling both structural and functional recovery.^69^

To quantify the self-healing efficiency, mechanical properties and electrical performance, they were compared before and after the healing process. In terms of electrical performance, the self-healing efficiency for conductivity (Fig. 7c) ranged between 82% and 92%, depending on the conductive component concentration. Interestingly, a slight decrease in electrical recovery was observed with increasing PANI–AgNP content. This may be explained by greater flexibility and rearrangement of conductive domains, supporting more efficient charge transport restoration post-damage because of the lower PANI–AgNP concentrations. The CV curves (Fig. 7d) obtained after self-healing were largely consistent with the original profiles, indicating preserved redox activity and electron transfer capability across the hydrogel matrix. Moreover, the mechanical test results demonstrated that the hydrogels retained over 95% of their original modulus values after healing (Fig. 7e), regardless of the PANI–AgNP concentration. In the self-healed samples, a slight decrease in elastic modulus was observed, while the ultimate compressive strength (Fig. 7f) increased, particularly in samples with high conductive particle content. This can be explained by the reorganization of the polymer–filler network during the healing process, which may make the material slightly more compliant in the initial elastic region, while simultaneously enhancing particle bridging and interfacial interactions. As a result, the material exhibits improved load-bearing capacity at higher strains despite the minor reduction in modulus.

Consequently, the obtained self-healing efficiency values, which exceeded 95% for mechanical modulus and ranged between 82 and 92% for electrical conductivity, are considered highly satisfactory and well within acceptable limits for functional materials in soft robotic applications.^70,71^ All these findings confirm that the GelMa/PANI–AgNP hydrogels possess robust self-healing functionality, not only in terms of physical integrity but also in preserving their key electrochemical and mechanical properties. This highlights the potential of the fabricated GelMa/PANI–AgNP hydrogels for long-term and dynamic use in advanced soft robotic systems.

### Fabrication of micro-scale GelMa/PANI–AgNP hydrogels by two-photon 3D laser printing

**Fig. 8:**
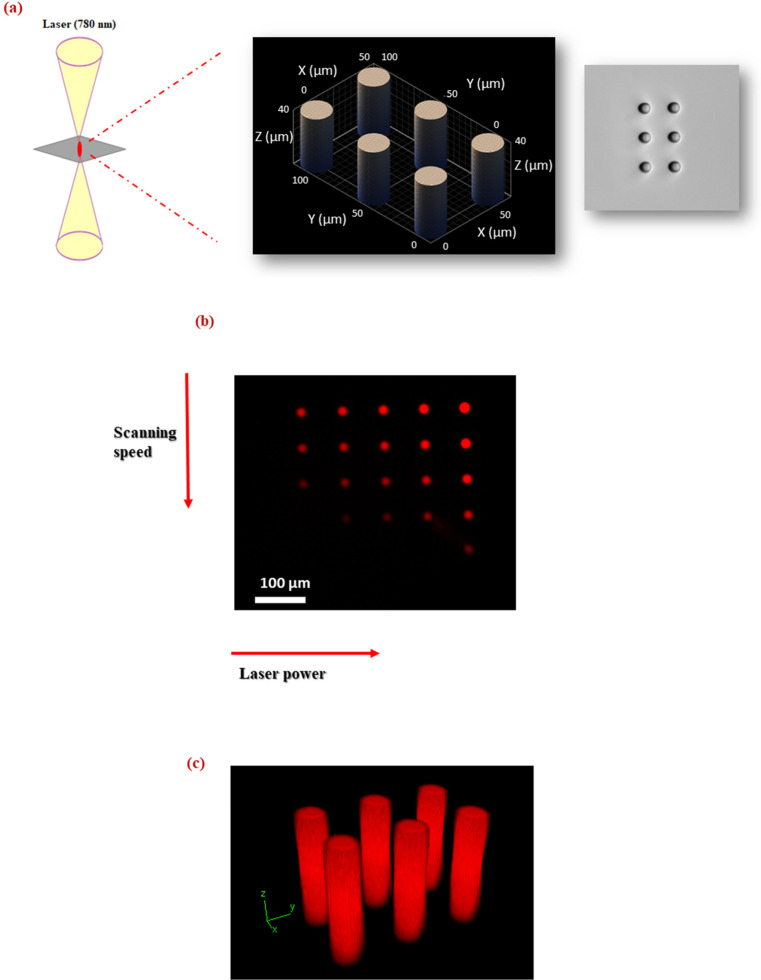
(a) Schematic representation of 2PP for the microfabrication of GelMa/PANI–AgNP hydrogels. (b) Two-photon imaging of micropillars containing rhodamine; micropillars were fabricated in a 5 × 5 array using a rhodamine-containing ink, with laser power varied along the *x*-axis and scanning speed varied along the *y*-axis. (c) 3D reconstructions of the micropillars. Imaging was performed using a femtosecond laser with a central wavelength of 1045 nm.

2PP enables the fabrication of complex three-dimensional hydrogel microstructures by utilizing a tightly focused femtosecond pulsed near-infrared laser in the presence of an appropriate photoinitiator (Fig. 8a). At the focal point, simultaneous absorption of two photons by the photoinitiator occurs, leading to localized excitation and subsequent radical generation. These radicals initiate a free-radical polymerization reaction confined to the focal volume, thereby inducing spatially controlled crosslinking within the precursor hydrogel solution. By precisely scanning the laser focal spot throughout the volume of the material, intricate 3D hydrogel geometries can be constructed. After printing, the non-crosslinked material is removed through a development step, leaving behind the desired micro-scale hydrogel structures.

In order to achieve optimal printability and structural stability for the prepared photoresist, a comprehensive dual-optimization strategy was implemented *via* (i) formulation of the photo-crosslinkable hydrogel ink and (ii) fine-tuning of the 2PP printing parameters. The first stage of optimization focused on the hydrogel formulation to ensure both photoreactivity and structural integrity for high-resolution microfabrication by investigating key parameters such as GelMa and PANI–AgNP concentrations. Different concentrations of GelMa were tested for the optimization. Lower concentrations led to insufficient structural stability and collapse of printed structures, while excessively high concentrations limited photo-crosslinking efficiency due to light attenuation and diffusion constraints. At the end, a GelMa concentration of 25% was found to provide the best balance between structural stability and photopolymerization performance and was selected as the optimum GelMa concentration for the photoresist formulation.

Since the optimization of the conductive component concentration is also critical for the success of 2PP of the ECHs, varying amounts of PANI–AgNPs were tried for the printing process. Preliminary tests demonstrated that formulations containing high concentrations of the PANI–AgNP composite exhibited significant light absorption at the near-infrared wavelength used for 2PP, resulting in rapid localized heating and micro-explosions. Such behavior is typically associated with non-uniform dispersion or local agglomeration of conductive nanoparticles, leading to excessive optical scattering and energy confinement. This excessive optical absorption likely hindered the penetration depth and led to an overexposure effect, disrupting voxel-level precision. Conversely, formulations with too low a concentration of PANI–AgNPs failed to initiate sufficient crosslinking under the same laser parameters. This is likely due to their reduced overall light absorption and potential quenching effects that diminished photoinitiator efficiency in the composite network. At 10% (v/v) PANI–AgNPs, better printed microstructures were obtained without inducing aggregation that would interfere with light propagation during printing. Therefore, this concentration was selected as the optimal value, as it provided a balance between effective photopolymerization and uniform structure formation in the microscale (*via* 2PP) and the same concentration value also showed superior electrical and mechanical performance in macro-scale hydrogels fabricated through UV crosslinking.

Following ink optimization, printing conditions such as laser power and scanning speed were systematically tuned to maximize resolution and structural integrity. The laser power (LP) scaling factor was varied from 60% to 100%, while the scanning speed (SS) ranged from 30 μm s^−1^ to 110 μm s^−1^. The results revealed a critical threshold behavior in the polymerization window, significantly influenced by both parameters. At laser power scaling factors below 60%, no stable or visible microstructures could be fabricated at any tested scanning speed, indicating that the energy dose delivered to the focal volume was insufficient to initiate polymerization. Starting at a 60% laser power scaling factor, successful structuring was obtained at a scanning speed of 30 μm s^−1^. This condition represented the lower boundary of effective photopolymerization, where the combined effects of energy deposition and exposure time were adequate to initiate crosslinking.

As the scanning speed increased, higher laser powers were required to achieve comparable structural resolution. This trend is attributed to the decreased exposure time per unit area at higher scanning speeds, leading to a reduced energy dose received by the photopolymerizable region. To counteract this, an increase in laser power was necessary to maintain a sufficient photon flux and ensure that the photoinitiator molecules absorbed enough energy to undergo two-photon excitation and generate reactive radicals for crosslinking. This inverse relationship between scanning speed and required laser power is consistent with prior reports, where it has been shown that faster scanning reduces the local exposure time, necessitating higher laser powers to achieve polymerization thresholds.^34,72^

Moreover, a particularly important observation emerged when comparing the photopolymerization behavior of pure GelMa and GelMa/PANI–AgNP micro-scale hydrogels. Pure GelMa hydrogels could be polymerized effectively at significantly lower laser powers. In contrast, the incorporation of PANI–AgNPs required increased laser intensities to achieve comparable printing conformity. This issue could be primarily attributed to the optical and chemical characteristics introduced by the conductive nanoparticles. The presence of PANI–AgNPs increases light absorption and scattering within the resin, which attenuates the laser beam and reduces the efficiency of 2PP at the focal volume. As a result, higher laser power is necessary to deliver sufficient energy for effective crosslinking. In addition, the conductive nanoparticles may interfere with photopolymerization kinetics by acting as radical scavengers or altering the local chemical environment, thus further inhibiting efficient polymer chain formation.^34^ These combined effects explain the need for higher laser powers in conductive formulations to maintain the desired structural resolution and crosslinking density.

All these findings underline the critical role of fine-tuning 2PP parameters in fabricating complex hydrogel microstructures, especially when conductive nanoparticles are present, which can further influence optical properties and polymerization efficiency. The fully optimized photoresist composed of 25% GelMa, 10% PANI–AgNPs, and 68 mM LAP exhibited excellent 2PP compatibility. Structures fabricated using this formulation demonstrated high structural resolution, reproducibility, and stability, making them well-suited for applications requiring microscale electroactive components. This ability to precisely fabricate GelMa/PANI–AgNP conductive hydrogels at the microscale opens promising avenues for soft microrobotic systems in the future, such as microgrippers, microneedles, or flexible biosensors that demand both mechanical adaptability and electrical functionality.

Two-photon imaging was employed to observe the 3D imaging capability and structural integrity of micropillars fabricated using a rhodamine-doped photoresist. As depicted in Fig. 8, the micropillar arrays (5 × 5) were generated by systematically varying the laser power along the *x*-axis and the scanning speed along the *y*-axis (Fig. 8b). These variations enabled the investigation of optimal fabrication conditions for achieving consistent pillar structure and brightness.

Three-dimensional reconstructions of the micropillars (Fig. 8c) revealed clear structural definition, indicating successful polymerization. The observed fluorescence intensity correlated strongly with the fabrication parameters: micropillars formed at higher laser powers and lower scanning speeds exhibited increased brightness, consistent with higher exposure doses resulting in more efficient photopolymerization and better fluorophore entrapment. Conversely, at lower laser powers and higher speeds, a reduced signal was observed, potentially due to insufficient crosslinking or incomplete excitation of embedded rhodamine. The successful visualization of these structures under two-photon excitation confirms that rhodamine remains photo-stable and active post-polymerization, making it a suitable internal marker for non-destructive 3D imaging and future functionalization studies.

The autofluorescence behavior of the fabricated micropillar arrays was systematically analyzed at two different excitation wavelengths (488 nm and 561 nm) to evaluate their intrinsic fluorescence properties, which is crucial for further downstream bio-imaging applications. Representative autofluorescence images are provided in the SI (Fig. S4).

## Conclusion

In this study, a multifunctional GelMa-based ECH system was developed for potential applications in soft material applications. The system was fabricated using complementary fabrication techniques at the macro- and microscale, with PANI–AgNP composites providing a dual conduction mechanism that ensured both sufficient electrical conductivity and homogeneous particle distribution. Comprehensive chemical, electrical, mechanical, and biocompatibility characterization studies confirmed the hydrogel's suitability for biomedical and soft robotic applications. Importantly, the material demonstrated self-healing capability, enabling autonomous recovery of both structural integrity and electrical functionality after damage. High-resolution 2PP allowed successful micro-scale fabrication, highlighting the system's versatility and scalability. To our knowledge, this is the first report combining macro-UV crosslinking and micro-2PP fabrication in a GelMa/PANI–AgNP platform, integrating self-healing, biocompatibility, and conductivity across multiple length scales. This work establishes a versatile platform for adaptive, durable, and miniaturized soft materials, providing a foundation for future studies aimed at developing conductive materials for both biomedical and soft robotic technologies.

## Conflicts of interest

There are no conflicts to declare.

## Data Availability

The data that support the findings of this study are available on heiDATA (https://heidata.uni-heidelberg.de and through https://doi.org/10.11588/DATA/6SWFRH).

## References

[cit1] Lee Y., Song W., Sun J.-Y. (2020). Mater. Today Phys..

[cit2] Rus D., Tolley M. T. (2015). Nature.

[cit3] Jia M., Rolandi M. (2020). Adv. Healthcare Mater..

[cit4] López-Díaz A., Vázquez A. S., Vázquez E. (2024). ACS Nano.

[cit5] Jiao D., Zhu Q. L., Li C. Y., Zheng Q., Wu Z. L. (2022). Acc. Chem. Res..

[cit6] Siemsen K., Rajput S., Rasch F., Taheri F., Adelung R., Lammerding J., Selhuber-Unkel C. (2021). Adv. Healthcare Mater..

[cit7] Liu J., Gao Y., Lee Y.-J., Yang S. (2020). Trends Chem..

[cit8] Shen Z., Chen F., Zhu X., Yong K.-T., Gu G. (2020). J. Mater. Chem. B.

[cit9] Barwig C., Colaco R., Koch A. S., Geiger S., Curticean E. R., Wacker I., Wang Z., Schmidt M., Sonn A., Pashapour S. (2025). Adv. Intell. Syst..

[cit10] Barwig C., Sonn A., Spratte T., Mishra A., Blasco E., Selhuber-Unkel C., Pashapour S. (2024). Adv. Intell. Syst..

[cit11] Colombo F., Taale M., Taheri F., Villiou M., Debatin T., Dulatahu G., Kollenz P., Schmidt M., Schlagheck C., Wittbrodt J. (2024). Adv. Funct. Mater..

[cit12] Shang Y., Wang J., Ikeda T., Jiang L. (2019). J. Mater. Chem. C.

[cit13] Spratte T., Arndt C., Wacker I., Hauck M., Adelung R., Schröder R. R., Schütt F., Selhuber-Unkel C. (2022). Adv. Intell. Syst..

[cit14] Ko J., Kim C., Kim D., Song Y., Lee S., Yeom B., Huh J., Han S., Kang D., Koh J.-S. (2022). Sci. Robot.

[cit15] Li J., Cao J., Lu B., Gu G. (2023). Nat. Rev. Mater..

[cit16] Ohm Y., Liao J., Luo Y., Ford M. J., Majidi C. (2023). Adv. Mater..

[cit17] Zhou C., Wu T., Xie X., Song G., Ma X., Mu Q., Huang Z., Liu X., Sun C., Xu W. (2022). Eur. Polym. J..

[cit18] Park C. S., Kang Y.-W., Na H., Sun J.-Y. (2024). Prog. Polym. Sci..

[cit19] Lv Z., Li W., Wei J., Ho F., Cao J., Chen X. (2023). CCS Chem..

[cit20] Kougkolos G., Golzio M., Laudebat L., Valdez-Nava Z., Flahaut E. (2023). J. Mater. Chem. B.

[cit21] Lang C., LaNasa J. A., Utomo N., Xu Y., Nelson M. J., Song W., Hickner M. A., Colby R. H., Kumar M., Hickey R. J. (2019). Nat. Commun..

[cit22] Li T., Liang B., Ye Z., Zhang L., Xu S., Tu T., Zhang Y., Cai Y., Zhang B., Fang L. (2022). Biosens. Bioelectron..

[cit23] Khazaeli A., Godbille-Cardona G., Barz D. P. (2020). Adv. Funct. Mater..

[cit24] Liu L., Yang B., Wang L.-Q., Huang J.-P., Chen W.-Y., Ban Q., Zhang Y., You R., Yin L., Guan Y.-Q. (2020). J. Mater. Chem. B.

[cit25] Yang Y., Jiao P. (2023). Mater. Today Adv..

[cit26] Arndt C., Hauck M., Wacker I., Zeller-Plumhoff B., Rasch F., Taale M., Nia A. S., Feng X., Adelung R., Schroder R. R., Schutt F., Selhuber-Unkel C. (2021). Nano Lett..

[cit27] Aycan D., Karaca F., Alemdar N. (2023). Mater. Today Commun..

[cit28] Aycan D., Karaca F., Koca A., Alemdar N. (2023). Int. J. Biol. Macromol..

[cit29] Tabrizian S. K., Terryn S., Vanderborght B. (2025). Adv. Intell. Syst..

[cit30] Roels E., Terryn S., Iida F., Bosman A. W., Norvez S., Clemens F., Van Assche G., Vanderborght B., Brancart J. (2022). Adv. Mater..

[cit31] Wu M., Han L., Yan B., Zeng H. (2023). Supramol. Mater..

[cit32] Wang X., Bai Z., Zheng M., Yue O., Hou M., Cui B., Su R., Wei C., Liu X. (2022). J. Sci.: Adv. Mater. Devices.

[cit33] Kröger F., Eichelmann R., Sauter G., Pollien A., Tegeder P., Gade L. H., Blasco E. (2024). RSC Appl. Polym..

[cit34] Lichade K. M., Shiravi S., Finan J. D., Pan Y. (2024). Addit. Manuf..

[cit35] Spratte T., Geiger S., Colombo F., Mishra A., Taale M., Hsu L. Y., Blasco E., Selhuber-Unkel C. (2023). Adv. Mater. Technol..

[cit36] Lin Y., Yang R., Wu X. (2023). RSC Appl. Polym..

[cit37] Ng W. W., Chen W.-H., Thiam H. S., Lim S., Chih Y.-K., Pang Y. L. (2026). RSC Adv..

[cit38] Farasatkia A., Kharaziha M., Ashrafizadeh F., Salehi S. (2021). Mater.
Sci. Eng., C.

[cit39] Ruiz C., Vera M., Rivas B. L., Sánchez S., Urbano B. F. (2020). RSC Adv..

[cit40] Duymaz D., Karaoğlu İ. C., Kizilel S. (2025). Macromol. Rapid Commun..

[cit41] Alexa R. L., Iovu H., Ghitman J., Serafim A., Stavarache C., Marin M.-M., Ianchis R. (2021). Polymers.

[cit42] Badi N., Khasim S., Pasha A., Alatawi A. S., Lakshmi M. (2020). J. Bio- Tribo-Corros..

[cit43] Haque S. U., Inamuddin, Nasar A., Rajender B., Khan A., Asiri A. M., Ashraf G. M. (2017). Sci. Rep..

[cit44] Gizdavic-Nikolaidis M. R., Pupe J. M., Jose A., Silva L. P., Stanisavljev D. R., Svirskis D., Swift S. (2023). Synth. Met..

[cit45] Tan X., Wang J., Pang X., Liu L., Sun Q., You Q., Tan F., Li N. (2016). ACS Appl. Mater. Interfaces.

[cit46] Fatema U. K., Rahman M. M., Islam M. R., Mollah M. Y. A., Susan M. A. B. H. (2018). Macromol. Symp..

[cit47] Gasaymeh S. S., Almansoori N. N. (2020). Results Phys..

[cit48] Zhang S., Wang C. (2023). Nano-Struct. Nano-Objects.

[cit49] Wu Y., Chen Y. X., Yan J., Quinn D., Dong P., Sawyer S. W., Soman P. (2016). Acta Biomater..

[cit50] Sun Z., Ou Q., Dong C., Zhou J., Hu H., Li C., Huang Z. (2024). Exploration.

[cit51] Schuurmans C. C., Brouwer A. J., Jong J. A., Boons G.-J. P., Hennink W. E., Vermonden T. (2021). ACS Omega.

[cit52] Yu H., Luo X., Li Y., Shao L., Yang F., Pang Q., Zhu Y., Hou R. (2024). Polymers.

[cit53] Wang R., Cheng C., Wang H., Wang D. (2024). ChemPhysMater.

[cit54] Ifergan-Azriel L., Bar-Am O., Saar G., Cohen T., Loebel C., Burdick J. A., Seliktar D. (2025). ACS Biomater. Sci. Eng..

[cit55] Ishikawa S., Iijima K., Matsukuma D., Iijima M., Osawa S., Otsuka H. (2021). Mater. Today Adv..

[cit56] Rossi F., Santoro M., Casalini T., Veglianese P., Masi M., Perale G. (2011). Int. J. Mol. Sci..

[cit57] Van Tomme S. R., van Nostrum C. F., de Smedt S. C., Hennink W. E. (2006). Biomaterials.

[cit58] Calvert P. (2009). Adv. Mater..

[cit59] Su T., Xu M., Lu F., Chang Q. (2022). RSC Adv..

[cit60] Aldana A. A., Malatto L., Rehman M. A. U., Boccaccini A. R., Abraham G. A. (2019). Nanomaterials.

[cit61] Bryning M. B., Milkie D. E., Islam M. F., Hough L. A., Kikkawa J. M., Yodh A. G. (2007). Adv. Mater..

[cit62] Chen Y., Xie Y. (2019). Adv. Electron. Mater..

[cit63] Haque S. U., Nasar A., Inamuddin, Rahman M. M. (2020). Sci. Rep..

[cit64] Ansari A. R., Ansari S. A., Parveen N., Ansari M. O., Osman Z. (2021). Materials.

[cit65] Correa C. M., Faez R., Bizeto M. A., Camilo F. F. (2012). RSC Adv..

[cit66] Zhu T., Ni Y., Biesold G. M., Cheng Y., Ge M., Li H., Huang J., Lin Z., Lai Y. (2023). Chem. Soc. Rev..

[cit67] Buaksuntear K., Limarun P., Suethao S., Smitthipong W. (2022). Int. J. Mol. Sci..

[cit68] Wang C., Huo S., Ye G., Zhang Q., Cao C.-F., Lynch M., Wang H., Song P., Liu Z. (2024). Chem. Eng. J..

[cit69] Liu D., Huyan C., Wang Z., Guo Z., Zhang X., Torun H., Mulvihill D., Xu B. B., Chen F. (2023). Mater. Horiz..

[cit70] Li X., Huang X., Mutlu H., Malik S., Theato P. (2020). Soft Matter.

[cit71] Wang J., Sawut A., Simayi R., Song H., Jiao X. (2024). J. Mech. Behav. Biomed. Mater..

[cit72] Harinarayana V., Shin Y. (2021). Opt. Laser Technol..

